# Diagnostic Challenges of Axenfeld-Rieger Syndrome and a Novel FOXC1 Gene Mutation in a Polish Family

**DOI:** 10.3390/jcm13195761

**Published:** 2024-09-27

**Authors:** Bogumił Wowra, Marzena Wysocka-Kosmulska, Karolina Stanienda-Sokół, Olga Łach-Wojnarowicz, Dariusz Dobrowolski, Edward Wylęgała

**Affiliations:** 1 Chair and Clinical Department of Ophthalmology, School of Medical Science in Zabrze, Medical University of Silesia in Katowice, Panewnicka 65 Str., 40760 Katowice, Poland; mwysocka554@gmail.com (M.W.-K.); sekretariat@wss5.pl (K.S.-S.); olga.lachwojnarowicz@gmail.com (O.Ł.-W.); dardobmd@wp.pl (D.D.); wylegala@gmail.com (E.W.); 2 Department of Ophthalmology, District Railway Hospital, Panewnicka 65, 40760 Katowice, Poland; 3 Department of Ophthalmology, St. Barbara Hospital, Trauma Centre, Medykow Square 1, 41200 Sosnowiec, Poland

**Keywords:** Axenfeld-Rieger syndrome, posterior embryotoxon, anterior segment dysgenesis, FOXC1 gene

## Abstract

(1) Axenfeld-Rieger syndrome (ARS) is a rare autosomal dominant disorder, the symptoms of which include both ocular and systemic abnormalities. In the studied subjects, the cornea was significantly opacified with peripheral scarring neovascularization, which is not specific to this syndrome. A suspicion of incorrect diagnosis was raised despite an initial diagnosis of a bilateral Chandler syndrome. (2) In order to provide the proper diagnosis, a DNA sequencing genetic test was conducted with three sisters carrying the presence of a genome imbalance in the FOXC1 gene. The aim of this study is to report a case of a Polish family with a novel gene mutation and its relation with ARS. (3) Our findings implicate the novel deletion of the FOXC1 gene in the pathogenesis of ARS in the affected family. The phenotypic variability observed, including differences in corneal and systemic anomalies, underscores the importance of genetic testing and suggests the influence of non-genetic factors on ARS manifestation.

## 1. Introduction

Anterior segment dysgenesis (ASD) disorders encompass a spectrum of rare developmental anomalies which affect the cornea, iris, and lens. Abnormalities of the anterior segment of the eye include, alone or combination, an anomalous structure of the iris that may be manifested as hypoplasia, pseudopolycoria, or corectopia, and malformed cornea with opacification or a posterior embryotoxon. Moreover, about half of patients with ASD suffer from glaucoma as a result of an iridocorneal angle involvement. Ocular findings may be separate or accompanied by systemic changes. The ASD spectrum includes Axenfeld-Rieger syndrome (ARS), aniridia, Peters anomaly, isolated trabeculodysgenesies as well as iridocorneal endothelial (ICE) syndrome. ICE consists of Chandler syndrome, Cogan-Reese syndrome and Essential iris atrophy [1,2,3].

Axenfeld Rieger syndrome (ARS), with a morbidity rate of 1/200,000, describes a group of hereditary heterogenous disorders of which the etiopathogenesis remains unexplained. The syndrome is inherited typically in autosomal dominant manner and is associated with mutations in genes FOXC1, PAX6, PITX2 [1]. It has been theorized that these genes play a pivotal role in the development of the neural crest origin in fetal life, which is abnormal in this condition.

The characteristic clinical ocular finding is embryotoxon posterior, which is a prominent Schwalbe’s line visible in the peripheral cornea. The iris is hypoplastic, with additional holes, pupil dislocation, and iris strands running across the angle of the anterior chamber. Corneal opacity is a rarely described manifestation. The first symptoms are noticed by parents early in their children. As this syndrome causes diagnostic problems due to considerable variation in the severity of ocular anomalies, it is necessary to pay attention to systemic abnormalities. The most common are dental and facial anomalies, which include hypertelorism, telecanthus, maxillary hypoplasia, microdontia (or characteristic conical shaped teeth), a reduced number of evenly spaced teeth (hypodontia), or even local deficiency of teeth (oligodontia, anodontia), as well as enamel abnormalities generating a large amount of caries at a young age. Further extra-ocular symptoms include the following: umbilical abnormalities, hypospadias, anal stenosis, pituitary and cardiac abnormalities, as well as SHORT syndrome (short stature, hyperextensible joints, hernia, ocular depression, Rieger anomaly, and teething delay) [4].

In contrast to ARS, Chandler syndrome is known to occur unilaterally at an adolescent age. Blurred vision and appearance of rainbow-colored circles around bright lights are a consequence of a corneal oedema in these subjects. What is interesting, and distinct from ARS, is that iris changes in Chandler syndrome are minimal and only affect the iris stroma, and so consequently the iris holes are not formed. Additionally, systemic symptoms are not observed [5].

Our study aims to report on a Polish family presenting with atypical features of ARS. We hypothesized that, in the studied patients, Chandler syndrome is not the correct diagnosis. Detailed, accurate analysis of clinical symptoms combined with genetic diagnostics allowed for detection of a novel mutation in the FOXC1 gene, thus far absent in the HGMD Professional 2024.1 Database, which confirmed ARS diagnosis. The clinical feature symptoms, both ocular and extra-ocular, of the affected patients are summarized.

## 2. Case Presentation

**Figure 1:**
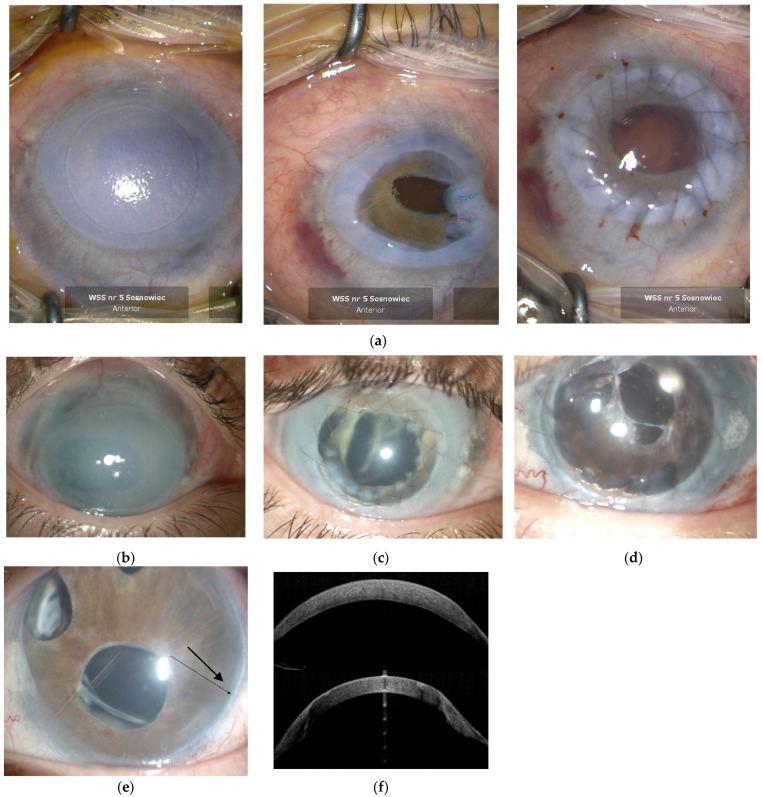
PKP revealed corectopia in the proband patient’s right eye (**a**). Slit lamp views show corneal opacity with peripheral scarring and neovascularization in the older sister before (**b**) and after PKP (**c**). The pseudopolycoria and hypotrophy of the iris in proband’s left eye (**d**). A posterior embryotoxon in the mother’s eye (black arrow) (**e**). Anterior Segment Optical Coherent Tomography scan of the older sister’s cornea before (above) and after (below) PKP (**f**) (compare to (**b**) and (**c**)).

A 35-year-old woman (proband) diagnosed with bilateral Chandler syndrome and congenital glaucoma was referred for a corneal treatment. Her visual acuity was determined by counting fingers in front of the right eye and hand motions in front of the left eye. Despite having undergone numerous anti-glaucoma surgeries (trabeculotomy, selective laser trabeculoplasty, the Ahmed Glaucoma Valve implantation, trabeculectomy), the intraocular pressure fluctuated. Due to a significant corneal opacity, oedema, peripheral scarring, and neovascularization in both eyes, the patient was qualified for a penetrating keratoplasty (PKP), which resulted in significant vision improvement; specifically, the BCVA (best-corrected visual acuity) in the right eye was 0.1, whereas in the left eye it was 0.06 six months after surgery (Figure 1a).

Accordingly, performing this medical procedure allowed for the further structures of the anterior segment of the eye to be visualized as follows: pseudopolycoria, corectopia and hypotrophy of the iris bilaterally (Figure 1d). The patient’s older sister, who was also diagnosed with bilateral Chandler syndrome and had comparable cornel symptoms, was qualified to undergo A PKP. Before the procedure, the right eye had only a sense of light (Figure 1b), and she had vision in the left eye to a distance of 20 cm; six months after the procedure, her right eye BCVA have been hand motion (Figure 1c,f), whereas in the left eye 0.06. The anterior segment of her eyes had been comparable to her younger sister (proband) (Figure 1d). It should be noted that their poor visual acuity arose from intraocular lens dislocation and glaucoma damage. Both the reciprocity of the ocular findings and family occurrence led to the suspicion that Chandler syndrome was not the correct diagnosis. To confirm our hypothesis, the patient’s mother and the youngest sister were examined. We discovered a posterior embryotoxon in the transparent cornea and iris anomalies in the mother’s only eye, the BCVA of which was 0.3 (Figure 1e). Her other eye was prosthetic; the reason for this is unknown. The third child, the youngest sister, despite good visual acuity (BCVA in the right eye was 0,6 and left eye 0.8), revealed clear cornea and iris abnormalities that are characteristic of ARS. Thus, all family members described had been diagnosed with glaucoma. However, the condition of their corneas varied greatly. The proband and her older sister showed significant corneal opacity, oedema, peripheral scarring, and neovascularization in both eyes. In contrast, the mother and the youngest sister had clear corneas. Afterwards, the diagnosis of Chandler syndrome was perspicuous; nevertheless, due to the doubtful corneal appearance the of daughters’ eyes, we decided to collect the material for genetic tests from the three sisters. Anterior Segment Optical Coherent Tomography scan of the older sister’s cornea shows difference between cornea structure before and after transplantation (Figure 1f).

**Figure 2:**
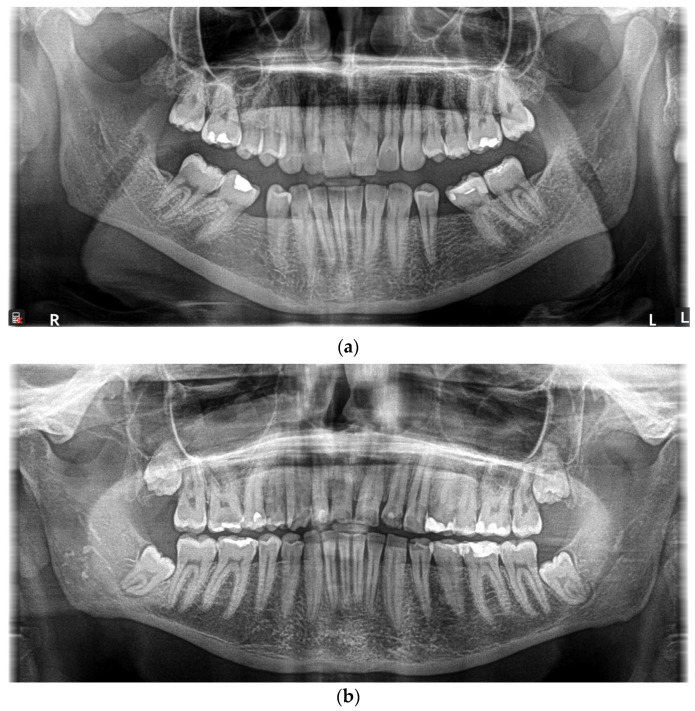
Panoramic ragiograph of the proband (**a**) revealed the lack of a premolar’s bud (hypodontia). The microdontia of incisors in the youngest sister was present (**b**).

The diagnosis of ARS was confirmed by a DNA sequencing genetic test in the sisters that indicated the presence of a genome imbalance in the form of a pathogenic extensive deletion covering a fragment of exon 1 and the entire sequence of the untranslated region 3′ (3′UTR) of the FOXC1 gene present in one allele of this gene (in a heterozygous system). It should be pointed out that a previously mentioned mutation had not yet been registered in the HGMD Professional 2024.1 Database. Notwithstanding the fact that the patients did not report any symptoms, our study revealed numerous ARS-specific changes. The panoramic radiograph (Figure 2) confirmed hypodontia as a lack of a premolar’s bud, microdontia of the incisors, as well as enamel hypoplasia and hipomineralization. A left anterior fascicular block and a bicuspidal aortic valve were discovered through a ECG and echocardiogram.

**Figure 3:**
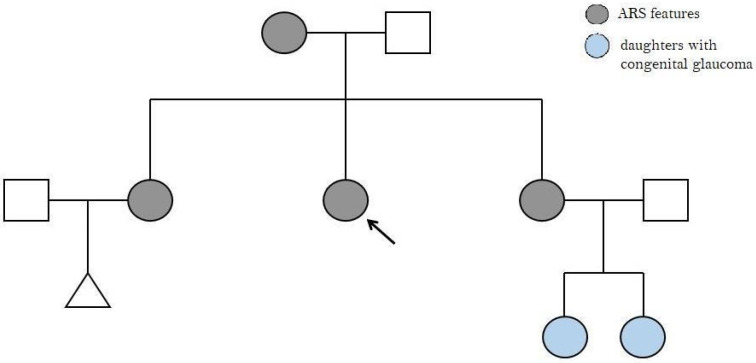
Pedigree of a Polish family affected by ARS with FOXC1 gene mutation. The arrow points out the proband. Squares indicate males, circles females, and the triangle indicates the miscarriage. Gray symbols represent affected individuals and blue symbols represent children with diagnosed congenital glaucoma. White symbols indicate unaffected individuals.

Other family members, including the father and two daughters of one of the sisters, were not available for examination. In addition, daughters of the oldest sister were diagnosed with congenital glaucoma (Figure 3). 

## 3. Discussion

For the first time in the literature, ARS was described by a German ophthalmologist in 1920. In 1934, Rieger extended the description by adding corectopia, polycoria, and systemic symptoms [4,6,7].

Both ARS and Chandler syndrome are still underexplored disorders and the subject of misdiagnosis, particularly in cases with atypical symptoms, such as those presented in this paper. In the literature, the presence of bilateral Chandler syndrome is uncommon. The first mentions of bilateral Chandler syndrome appeared in 1999 in works such as those by Ichhpujani P. et al., who reported a case of a 22-year-old female with a bilateral oedematic cornea [5,8].

Agarwal M. et al. described bilateral Chandler syndrome accompanying band keratopathy [9]. In contrast, Hong-shu Zhao et al. enrolled 10 people with a diagnosis of bilateral ICE syndrome, and, after a detailed examination, it was found that half of them had ARS [10]. Interestingly, none of studied patients had bilateral ICE syndrome. With reference to the etiology of both ARS and Chandler syndrome, and in the presented family case, a vigilant approach is suggested in making the final diagnosis. Due to the heterogeneity and high resemblance of both syndromes, genetic testing is the only test that provides diagnostic certainty.

The connection of ARS syndrome with FOXC1, PITX2 and PAX6 is well documented [11,12,13,14,15,16,17,18]. According to genetic etiology and phenotypic traits, we distinguished three types of ARS. Type 1 was induced by PITX2 gene mutation; the origin of type 2 is unknown, but our hypothesis connects this type with chromosome 13 and is presented in this study; type 3 is determined by FOXC1 gene mutation [19]. Currently, there are over 400 documented cases of individuals with heterozygous, disease-causing mutations, including whole gene deletions, missense, and nonsense variants in FOXC1 [20,21]. Forkhead Box C1 (FOXC1) is a leading transcription factor for neural crest and ocular evolution in which mutation conducts to anterior segment defects such as a lack of or a less-developed Schlemm’s canal, abnormal trabecular meshwork, iris hypoplasia, displaced irregular pupil and aberrant Schwalbe’s line. It was discovered that, in FOXC1 heterozygote mice genes, the iris stroma and iris pigment epithelium appeared to be flattened and thinner. These anomalies may contribute to glaucoma development [13,22,23]. Nevertheless, the etiology of glaucoma is not well defined, hence highlighting the need to identify the mechanism. One of the hypotheses indicates that ciliary body and drainage angle morphology may lead to increased IOP. It is also evidenced that the protection for the trabecular meshwork from impairments resulting in elevated IOP produces the antioxidate enzymes by the ciliary epithelium. It is possible that FOXC1 contributes to both the volume and composition of the aqueous humor. It should be noted that, compared to other mutations, ARS caused by FOXC1 remarkably promotes congenital glaucoma development. Similar results were published by Zhou et al. in their study proving a significant difference between PITX2 and FOXC1 in glaucoma [20]. In our study, it was not only the proband and examined family members but also the children of the oldest sister that were diagnosed with congenital glaucoma [14,24,25].

Furthermore, it was proven that FOXC1 mutation in mice may lead to disorganization of corneal stroma and endothelium. Deletion in the FOXC1 gene in mice remains a trigger for collagen structure disruption. The cornea of heterogenous FOXC1 mice revealed both a significant reduction and disorganization of collagen fibers, as well impaired corneal stroma cells [12]. Subjects of this case study displayed a significant opacification of the cornea. Although the pathogenesis of the corneal opacity in these subjects remains impartially understood, considering the above, we raised the suspicion that severely chaotic and thickened stroma resembled the structure of the sclera; therefore, the cornea in patients with a FOXC1 gene mutation may undergo sclerotization. Despite a different pathophysiology, pathological corneal neovascularization and ulcers have been also associated with this mutation [26]. We do not know what the patients’ cornea looked like in childhood, but it can be assumed that neovascularization is secondary and the haziness of the entire cornea was a primary feature. Significant anterior segment phenotypes consistent with FOXC1 disruption and corneal neovascularization were also noted in the patients described by Reis et al. [27]. Additionally, recent research indicates that mutations in FOXC1 are associated with a greater extent of corneal abnormalities and a higher frequency of glaucoma compared to mutations in PITX2 [28]. The transcription factor FOXC1 has a role in a pathological corneal vessel’s growth. In recent years, Seo at al. verified FOXC1 as the key inhibitor of corneal neovascularization in mice [29]. This protective mechanism is not attributed to the level of Vascular Endothelial Growth Factor (VEGF) but to the decreased bioavailability of VEGF triggered by reducing matrix metaloproteinases (MMP) in extracellular matrix (ECM). This is linked with disorganization of the EMC, thereby contributing to the impaired corneal transparency. Understanding the physiological mechanisms which protect the cornea from vascular growth is crucial not only for vision but also for novel therapy development.

FOXC1 gene mutations lead to various systemic complications beyond the ocular symptoms typical of ARS. These mutations affect multiple organ systems due to FOXC1’s role in embryogenesis and tissue development. Systemic manifestations can include cardiovascular abnormalities, such as septal defects, or more complex congenital heart issues and sensorineural hearing loss, indicating impaired auditory system development. Craniofacial anomalies, including midface hypoplasia, dental issues like microdontia, and skeletal abnormalities such as short stature further demonstrate the gene’s broad developmental impact. These findings underscore FOXC1’s crucial function across multiple bodily systems, providing insight into the diverse biological pathways it influences [30]. In contrast, in the subject family, no hearing loss was present.

There are numerous confirmed FOXC1 gene mutations, whereas our study reports a novel one. There is a diversity in gene variation outcomes with a heterogeneity of phenotypes in patients with ARS. Our study illustrates a family in which the same mutation in the FOXC1 gene was diagnosed, while the clinical picture between patients differed significantly. All family members were diagnosed with glaucoma, but the corneal condition differed dramatically, which may indicate that it is not only genetic factors that influence the clinical picture. The cause of corneal haze in ARS is still unknown and requires further investigation. Additionally, the basis of a difference in the phenotypes remains unclear. It is suggested that factors so far unknown may be the cause of the variety in the phenotypes in patients with the same mutations. The studied family proves how multiple clinical manifestations can be presented by one mutation. Our hypothesis was confirmed by examining other family members and by additional genetic tests that diagnosed Axenfeld-Rieger syndrome in the described patients.

## 4. Conclusions

Our study sheds light on the complex and varied nature of Anterior Segment Dysgenesis (ASD) disorders, particularly focusing on the misdiagnosis and clinical presentation of Axenfeld-Rieger syndrome (ARS) in a Polish family. By meticulously analyzing the clinical symptoms and conducting genetic diagnostics, we identified a novel mutation in the FOXC1 gene, thereby confirming the diagnosis of ARS.

The proband and her family members exhibited a range of ocular and systemic anomalies, including bilateral corneal opacity, glaucoma, and iris abnormalities, alongside systemic symptoms like dental anomalies and congenital heart issues. The discovery of a novel FOXC1 mutation underscores the genetic diversity and phenotypic variability within ARS. Our findings emphasize the critical role of genetic testing in accurately diagnosing ASD disorders, particularly in cases with atypical or overlapping symptoms. The presence of glaucoma and significant corneal abnormalities in the proband, contrasting with the clear corneas displayed by her mother and sister, highlights the phenotypic variability even within the same genetic mutation. This suggests that non-genetic factors may also influence the clinical manifestation of ARS.

Additional studies of the cornea in ARS may reveal the pathogenesis of haze formation, crucial for developing new therapies. Understanding the cellular dynamics, genetic mutations, and extracellular matrix alterations that lead to corneal haze can identify potential therapeutic targets. These insights could pave the way for treatments like gene therapy, anti-fibrotic drugs, and biomaterials to improve vision and quality of life for ARS patients.

The study also underscores the importance of recognizing systemic symptoms associated with ARS, as these can provide crucial diagnostic clues. The identified FOXC1 mutation links the disorder to broader developmental pathways, affecting multiple organ systems and contributing to the diverse clinical features that have been observed.

In conclusion, our research contributes to the understanding of ARS and ASD disorders by highlighting the importance of comprehensive clinical and genetic evaluations. The novel FOXC1 mutation identified in this study expands the genetic spectrum of ARS and emphasizes the need for further research into the mechanisms underlying phenotypic variability and systemic involvement in these disorders.

## Data Availability

The original contributions presented in the study are included in the article, further inquiries can be directed to the corresponding authors.
